# The Effect of Serum 25-Hydroxyvitamin D on Elevated Homocysteine Concentrations in Participants of a Preventive Health Program

**DOI:** 10.1371/journal.pone.0161368

**Published:** 2016-08-22

**Authors:** Truong-Minh Pham, John Paul Ekwaru, Silmara S. Mastroeni, Marco F. Mastroeni, Sarah A. Loehr, Paul J. Veugelers

**Affiliations:** Population Health Intervention Research Unit, School of Public Health, University of Alberta, Edmonton, Canada; Universidad Europea de Madrid, SPAIN

## Abstract

Both lower serum 25-hydroxyvitamin D [25(OH)D] and elevated homocysteine concentrations are potential risk factors for cardiovascular disease (CVD). A recent analysis of the National Health and Nutrition Examination Survey reported an inverse association of serum 25(OH)D with homocysteine, however, the longitudinal relationship has yet to be investigated. We hypothesized and examined whether a temporal increase in 25(OH)D concentrations is paralleled by a reduction in the risk for elevated homocysteine. We analyzed data of 4475 participants with repeated assessments of serum 25(OH)D and homocysteine concentrations who enrolled in a preventive health program that encourages vitamin D supplementation and monitors serum 25(OH)D and homocysteine concentrations. We defined elevated homocysteine as concentrations greater than 13 micromoles per liter. Logistic regression was applied to assess the association of temporal changes in serum 25(OH)D with the risk of elevated homocysteine. We observed an inverse gradient whereby greater increases in 25(OH)D concentrations were associated with a lower prevalence of elevated homocysteine. Relative to those without temporal increases in 25(OH)D, participants who showed improvements in their serum 25(OH)D concentrations of “<25”, “25–50”, “50–75”, and “≥75” nanomoles per liter at follow up were 0.92 (95% confidence interval: 0.62–1.37), 0.52 (0.33–0.80), 0.34 (0.20–0.58), and 0.32 (0.19–0.54) times as likely to have elevated homocysteine, respectively. These observations suggest that temporal improvements in vitamin D status reduce serum homocysteine concentrations, and therefore may potentially contribute to the primary prevention of CVD.

## Introduction

The hypothesis that elevated concentrations of homocysteine, a product of methionine metabolism [1], may be a risk factor for coronary artery disease was raised decades ago after arterial lesions were observed in children with high homocysteine concentrations [2, 3]. Since then, links between elevated homocysteine and cardiovascular outcomes have been reported [4–6].

Vitamin D is synthesized in the skin under the exposure of sunlight, and can be also obtained through the diet and supplementation. Circulating 25-hydroxyvitamin D [25(OH)D] is the established nutritional biomarker for vitamin D status [7]. Although the role of vitamin D in sustaining calcium homeostasis and bone health is well recognized [7, 8], vitamin D has also been suggested to have other health benefits such as the prevention of cardiovascular disease (CVD) [9]. Since both lower 25(OH)D concentrations and elevated homocysteine are potential risk factors for CVD, and because vitamin D may be involved in the regulation of the gene expression of enzymes involved in homocysteine metabolism, Amer and Qayyum hypothesized that vitamin D status may have an impact on serum homocysteine [10]. In their cross-sectional examination of data from the National Health and Nutrition Examination Survey (NHANES) the authors found a significant inverse association between vitamin D status and serum homocysteine concentrations, therefore confirming their hypothesis, though only for those with serum 25(OH)D concentrations below 52.5 nanomoles per liter.

Since the aforementioned analysis was conducted in a cross-sectional sample of observational data, we examined whether temporal improvements in vitamin D status may lead to reductions in homocysteine concentrations using longitudinal data from participants of a preventive health program. We hypothesized that a temporal increase in 25(OH)D concentration is paralleled by a decrease in the risk for elevated serum homocysteine concentrations.

## Materials and Methods

### Study population

The Pure North S’Energy Foundation (PN) in Calgary, Alberta, Canada, is a not-for-profit organization that offers a preventive health program, of which details have been described elsewhere [11, 12]. Briefly, the program was launched in October 2007 and employs health professionals, with the objective to provide informed lifestyle counseling to volunteer participants. At enrollment, participants complete lifestyle questionnaires, medical history, and biometric measurements taken (height, weight, waist circumference, blood pressure), and have blood drawn for the assessment of serum 25(OH)D and other biomarkers. The information collected is used to inform the health professionals as a basis for lifestyle counseling. Vitamin D supplementation is encouraged given Canada’s Northern latitude, which results in limited sunlight and therefore inadequate cutaneous synthesis of vitamin D. Follow up visits for health assessments and lifestyle counseling are scheduled annually. The primary objective of the PN program is lifestyle counseling and disease prevention rather than scientific research. However, the PN makes their data available, in anonymized form, to the University of Alberta for secondary data analysis. For that purpose, participants signed and granted written informed consent to allow their relevant information to be used for secondary data analysis. The ethical approval for the use of the PN data for research and scientific reporting was granted by the Human Research Ethics Board of the University of Alberta. Data from 4475 participants aged 18 years or older with at least two measures of both serum 25(OH)D concentrations and serum homocysteine concentrations (one each at baseline and the others at follow up visit(s) in order to examine the effect of temporal changes in 25(OH)D on homocysteine concentrations) were included in the current analysis.

### Laboratory measurements

Serum 25(OH)D concentrations were measured by DiaSorin using chemiluminescence immunoassays on an automated LIAISON platform, and results were expressed as nanomoles per liter (nmol/L). The inter-assay coefficient of variation (CV) for 25(OH)D measurements was 11%. Serum homocysteine concentrations were measured by the cyclic enzyme method that employs an enzymatic rate principle on an automated analyzer; results were expressed as micromoles per liter (μmol/L), and the inter-assay CV was 4%. Serum vitamin B_12_ concentrations, a confounding variable because vitamin B_12_ plays a key role in homocysteine metabolism, were measured by the chemiluminescent immunoassay procedure. The inter-assay CV was 4%, and the results were expressed as picomoles per liter (pmol/L). Measurements for serum concentrations of triglycerides, total cholesterol, and high-density lipoprotein (HDL) cholesterol were performed by automated Roche Cobas 8000 Modular Analyzer Series. Results were expressed as millimoles per liter (mmol/L). The inter-assay CV was 2% for triglycerides, 1.5% for total cholesterol, and 2% for HDL cholesterol. Low-density lipoprotein (LDL) cholesterol concentrations were calculated from total cholesterol, HDL cholesterol and triglycerides according to the following formula: LDL cholesterol = Total cholesterol–HDL cholesterol–(Triglycerides /2.2).

### Assessment of other variables

We obtained information on age, sex, and smoking habits (categorized as never smoker, past smoker, and current smoker) from questionnaires completed by the participants. Participants were also asked to indicate how many glasses of alcohol containing beverages they consumed in a typical week. We categorized them as ‘non-drinker’ if they reported to consume less than two glasses per week and as ‘drinker’ when reporting two or more glasses per week. Participants were further asked to list their light, moderate, and vigorous activities for a typical week. We estimated the metabolic equivalent of task (MET) for each activity and multiplied this with the time performing these activities [13]. The weeks total (MET*hours per week) was categorized as low (MET*hours per week < 10), moderate (MET*hours per week between 10 and 20) and high (MET*hours per week > 20). Changes in physical activity were calculated by subtracting the baseline from the follow up and grouped as: ‘no improvement’, ‘moderate improvement’ (increase of < 10 MET*hours per week), ‘high improvement’ (increase of ≥10 MET*hours per week) [13]. Body mass index (BMI) was calculated from measured weight in kilograms divided by the square of the measured height in meters (kg/m^2^). Hypertension was defined as blood pressure equal to or greater than 140/90 mmHg, or if a self-reported antihypertensive medication was currently being taken.

### Statistical analyses

Elevated homocysteine status was defined as serum homocysteine concentrations >13 μmol/L according to the 95th percentile of distribution of the most comprehensive national survey to date among Canadians, the Canadian Health Measures Survey [14]. In a sensitivity analysis, we considered sex specific thresholds, 14.2 μmol/L for women and 15.2 μmol/L for men. These thresholds were derived from the baseline observations of this study and represent the sex specific 95^th^ percentiles. Baseline serum 25(OH)D concentrations were categorized as the following: “<50”, “50-<75”, “75-<100”, “100-<125”, and “≥125” nmol/L. The group with the lowest serum 25(OH)D concentrations (<50 nmol/L) was considered the reference group. Temporal changes in serum homocysteine and 25(OH)D concentrations were calculated by subtracting baseline concentrations from the follow up concentrations. Changes in 25(OH)D concentrations were classified into the following categories: “No improvement” (for values ≤ 0), “Increase of < 25”, “Increase of 25-<50”, “Increase of 50-<75”, and “Increase of ≥75” nmol/L. Cut-offs for vitamin B_12_ deficiency (< 148 pmol/L) and insufficiency (148-≤220 pmol/L) were established according to the definitions used by the CHMS [14]. Since only 41 participants (<1%) were vitamin B_12_ deficient, we combined those who were deficient and insufficient. Serum LDL cholesterol concentrations of ≥ 2.6 mmol/L were considered as elevated LDL levels [15]. Descriptive statistics were presented for both baseline and follow up values of serum 25(OH)D concentrations, serum homocysteine concentrations, and potential confounders. As various participants had more than one follow up visit, we used the most recent follow up visit when presenting these descriptive statistics; however, we used all follow up information in all longitudinal analyses. Cross-sectional associations of baseline homocysteine concentrations with 25(OH)D concentrations were analyzed using linear regression methods and expressed as differences in homocysteine (μmol/L) per 25 nmol/L difference in 25(OH)D concentrations. These analyses were adjusted for sex, age (per 10 years), body mass index, hypertension, serum LDL-cholesterol, smoking status, alcohol status, physical activity and vitamin B_12_ status. Linear regression was also applied to quantify the association between temporal changes in 25(OH)D and coinciding changes in homocysteine. This was applied for the range of 0 to 75 nmol/L in the temporal increase of 25(OH)D concentrations as the association of change in homocysteine and change in serum 25(OH)D was linear for this range. Logistic regression was applied to quantify the longitudinal relationship of 25(OH)D at baseline and changes in 25(OH)D during follow up with elevated homocysteine at follow up. The latter regression analyses were adjusted for the presence of elevated homocysteine at baseline, sex, baseline age (per 10 years), body mass index, hypertension, LDL cholesterol, smoking, drinking status, physical activity and changes in physical activity from baseline to follow up, and vitamin B_12_ status at baseline and at follow up. Logistic regression analyses were performed using generalized estimating equations methods to accommodate for the fact that some participants had more than one follow up visit. Missing values of for confounding variables were considered as missing categories in all multivariate regression analyses. As the substantial numbers of participants had missing information on vitamin B_12_ we repeated the regression analyses for the subgroup with complete information on vitamin B_12_.

Statistical analyses were performed using SAS 9.4 (SAS Institute Inc., Cary, USA). All statistical tests were two-sided and the level of statistical significance was at 0.05.

## Results

Participant characteristics (n = 4475) at baseline and follow up are presented in Table 1. There were 3250 (73%) participants with one follow up visit, 686 (15%) with two follow up visits, and 539 (12%) with three or more follow up visits. The mean and median serum 25(OH)D increased from 85 and 77 nmol/L at baseline to 121and 113 nmol/L at follow up, respectively. Of all participants, 1778 (40%) supplemented with vitamin D at baseline and 3534 (79%) at follow up. The median dose (IQR, interquartile range) was 3000 (1700–5000) international units at baseline and 7000 (4700–10000) at follow up. The mean and median serum homocysteine decreased from 9.8 and 9.3 μmol/L at baseline to 8.5 and 8.2 μmol/L at follow up, respectively. Elevated homocysteine concentrations (>13 μmol/L) were observed in 11.0% of participants at baseline and in 4.5% of participants at follow up. Serum vitamin B_12_ concentrations less than 220 pmol/L was observed in 8.4% of participants at baseline, and 2.7% of participants at follow up. The number of participants that participated in a high level of physical activity increased from 30% to 33%. Other potential confounders did not differ substantially from baseline to follow up (Table 1). The median time between baseline and follow up was 1.1 year.

**Table 1:** Baseline and follow up characteristics of 4475 study participants.

	Women (n = 2094)		Men (n = 2381)		Both sexes (4475)	
	Baseline	Follow up	Baseline	Follow up	Baseline	Follow up
**Serum 25(OH)D, nmol/L**						
Mean (SD)	93 (42)	124 (47)	78 (41)	118 (53)	85 (42)	121 (50)
Median (IQR)	86 (65–112)	118 (91–150)	70 (50–96)	110 (79–147)	77 (57–105)	113 (85–149)
**Serum homocysteine, μmol/L**						
Mean (SD)	9.2 (3.7)	7.7 (2.4)	10.4 (2.9)	9.2 (2.5)	9.8 (3.4)	8.5 (2.6)
Median (IQR)	8.7 (7.3–10.4)	7.3 (6.2–8.7)	9.9 (8.5–11.5)	8.9 (7.6–10.3)	9.3 (7.9–11.1)	8.2 (6.8–9.7)
95% percentile	14.2	11.6	15.2	13.5	14.8	12.8
**Elevated homocysteine, n (%)**^a^	173 (8.3)	55 (2.3)	298 (12.5)	145 (6.1)	471 (11.0)	200 (4.5)
**Age, mean (SD)**	50 (16)	51 (16)	46 (14)	48 (14)	48 (15)	49 (15)
**BMI, n (%)**^b^						
<18.5	36 (2)	42 (2)	6 (<1)	6 (<1)	42 (1)	48 (1)
18.5–<25.0	916 (44)	910 (44)	500 (22)	484 (21)	1416 (32)	1394 (32)
25.0–<30.0	612 (29)	618 (30)	1060 (46)	1066 (46)	1672 (38)	1684 (38)
> = 30.0	509 (25)	503 (24)	750 (32)	760 (33)	1,259 (29)	1263 (29)
Missing	21	21	65	65	86	86
**Hypertension, n (%)**^c^						
Normal	1623 (83)	1624 (85)	1637 (75)	1596 (75)	3260 (79)	3220 (80)
Elevated	338 (17)	286 (14)	550 (25)	536 (25)	888 (21)	822 (20)
Missing	133	184	194	249	327	433
**Serum LDL-cholesterol, n (%)**^d^						
Normal	811 (40)	792 (39)	735 (33)	710 (32)	1546 (36)	1502 (35)
Elevated	1199 (60)	1247 (61)	1523 (67)	1530 (68)	2722 (64)	2777 (65)
Missing	84	55	123	141	207	196
**Vitamin B12 status, n (%)**^b^^,^ ^e^						
Deficient/Insufficient	158 (8.9)	53 (2.6)	118 (7.9)	62 (2.7)	276 (8.4)	115 (2.7)
Adequate	1609 (91.1)	2002 (97.4)	1384 (92.1)	2205 (97.3)	2993 (91.6)	4207 (97.3)
Missing	327	39	879	114	1206	153
**Tobacco smoking status, n (%)**^b^						
Never smoker	929 (61)	598 (60)	628 (50)	653 (52)	1557 (56)	1251 (55)
Past smoker	443 (29)	289 (29)	336 (27)	322 (25)	779 (28)	611 (27)
Current smoker	160 (10)	116 (11)	290 (23)	294 (23)	450 (16)	410 (18)
Missing	562	1091	1127	1112	1689	2203
**Alcohol drinking status, n (%)**^b^						
Non-drinker	706 (49)	737 (52)	483 (38)	575 (35)	1189 (44)	1312 (43)
Drinker	732 (51)	682 (48)	778 (62)	1067 (65)	1510 (56)	1749 (57)
Missing	656	675	1120	739	1776	1414
**Physical activity, n (%)**^b^						
Low	679 (45)	555 (41)	442 (36)	461 (32)	1121 (40)	1016 (36)
Moderate	440 (29)	414 (30)	373 (30)	435 (31)	813 (30)	849 (30)
High	397 (26)	396 (29)	414 (34)	535 (37)	811 (30)	931 (33)
Missing	578	729	1152	950	1730	1679

25(OH)D, 25-hydroxyvitamin D; LDL-cholesterol, low-density lipoprotein cholesterol; nmol/L, nanomoles per liter; SD, standard deviation; IQR, interquartile range; μmol/L, micromoles per liter.

^a^ Elevated homocysteine was defined as serum concentrations >13 μmol/L.

^b^ Percentage for these variables do not include missing observations.

^c^ Hypertension was defined as blood pressure ≥140/90 mm Hg, or a self-report of taking antihypertensive medications;

^d^ Elevated LDL-cholesterol was defined as LDL-cholesterol concentration ≥2.6 mmol/L.

^e^ Vitamin B_12_ adequacy was defined as serum concentrations >220 picomoles per liter.

The cross-sectional associations between 25(OH)D and homocysteine concentrations at baseline are presented in Table 2. When all 4475 participants were considered, multivariable linear regression revealed that baseline homocysteine concentrations were 0.182 μmol/L lower for each 25 nmol/L difference in baseline 25(OH)D concentration. When the same analyses were repeated separately by subgroup of baseline 25(OH)D, we observed that with each 25 nmol/L difference in 25(OH)D, homocysteine concentrations were 1.056 μmol/L lower in those with 25(OH)D <50 nmol/L and 0.150 μmol/L lower in those with 25(OH)D ≥50 nmol/L (Table 2).

**Table 2:** Cross sectional associations of serum homocysteine and 25(OH)D concentrations based on baseline observations of 4475 study participants.

	Entire population(n = 4475)		25(OH)D <50 nmol/L(n = 809)		25(OH)D ≥50 nmol/L(n = 3666)	
	β (95% CI)	p	β (95% CI)	p	β (95% CI)	p
Univariate	-0.195 (-0.245; -0.145)	<0.01	-0.979 (-1.607; -0.350)	<0.01	-0.171 (-0.227; -0.115)	<0.01
Model 1	-0.212 (-0.261; -0.162)	<0.01	-1.090 (-1.692; -0.487)	<0.01	-0.167 (-0.220; -0.115)	<0.01
Model 2	-0.182 (-0.228; -0.136)	<0.01	-1.013 (-1.601; -0.425)	<0.01	-0.148 (-0.197; -0.100)	<0.01

25(OH)D, 25-hydroxyvitamin D; nmol/L, nanomoles per liter; β (95% CI), beta coefficient with 95% confidence interval. The β coefficients represent the difference in homocysteine concentration in μmol/L per 25 nmol/L difference in 25(OHD) concentrations. Model 1: adjusted for sex, age (per 10 years), body mass index, hypertension, serum LDL-cholesterol, smoking status, alcohol status, and physical activity. Model 2: adjusted for the same covariates as model 1, and additionally adjusted for serum vitamin B_12_ status.

**Fig 1:**
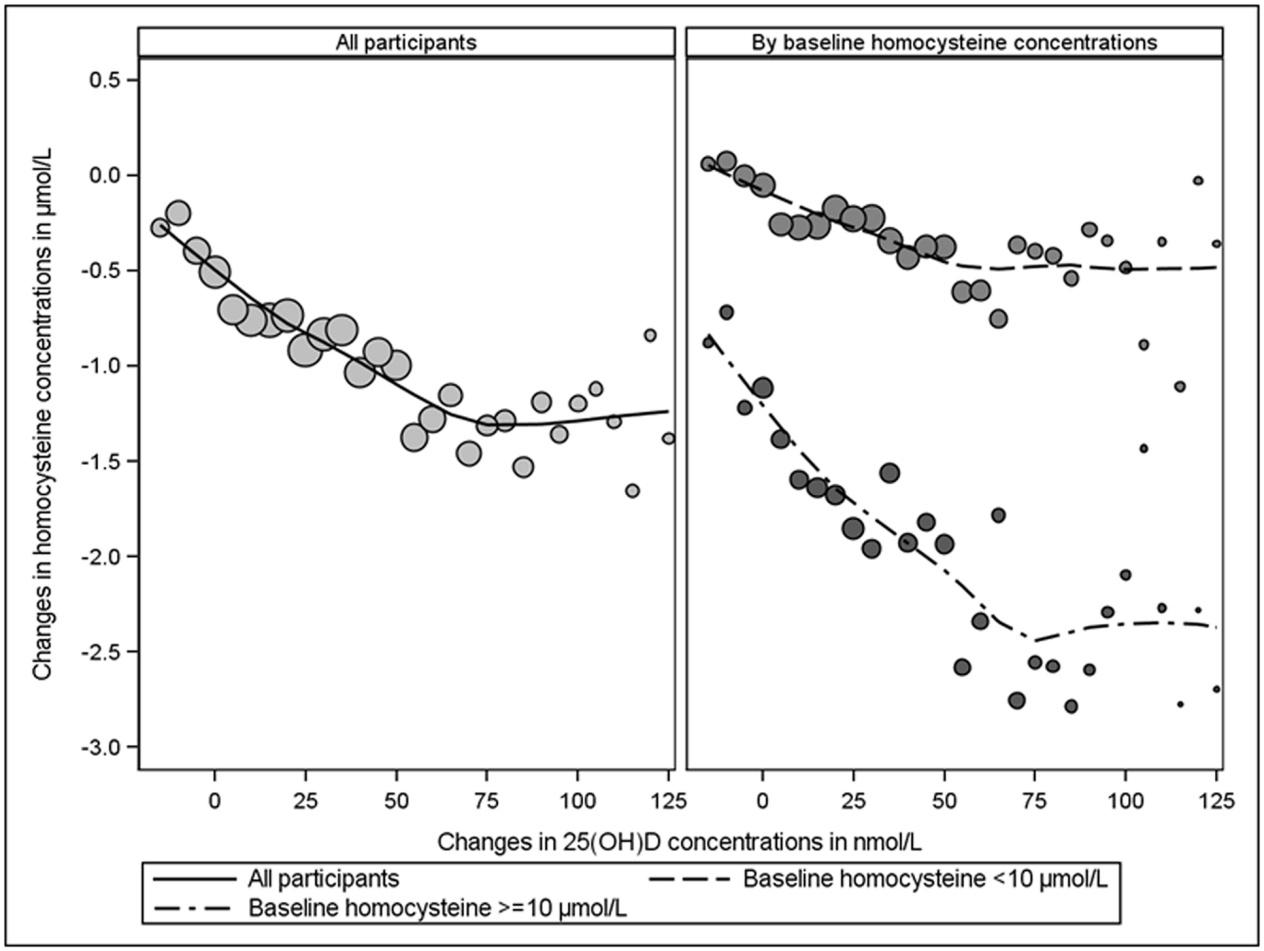
Associations of changes in serum 25(OH)D concentrations with coinciding changes in homocysteine. μmol/L denotes micromoles per liter; and nmol/L denotes nanomoles per liter.

Fig 1 depicts the associations between the temporal change in 25(OH)D concentrations and coinciding change in homocysteine. The left panel of the figure depicts this for all 4475 participants and reveals a gradual drop in homocysteine concentrations with increases in 25(OH)D concentrations up to increases of approximately 75 nmol/L beyond which homocysteine concentrations did not further drop. The right panel of the figure reveals that the association between change in homocysteine and change in 25(OH)D concentrations is more pronounced for participants with high baseline homocysteine concentrations. The linear regression of this association, presented in Table 3, confirms this and shows that the association is independent of sex, age, body mass index, hypertension, serum LDL-cholesterol, smoking status, alcohol status, physical activity and changes in physical activity, and serum vitamin B_12_ status and changes vitamin B_12_ status. In this regard, for every 25 nmol/L increase in 25(OH)D between baseline and follow up, the homocysteine concentration was lowered by an average of 0.15 μmol/L (Table 3). Repeating this analysis while excluding participants with missing information on vitamin B_12_ status, revealed a similar average of -0.17 μmol/L and a 95% confidence interval -0.22 to -0.11.

**Table 3:** Associations of change in serum homocysteine and coinciding change in serum 25(OH)D concentrations.

	All observations		Baseline homocysteine < 10 μmol/L		Baseline homocysteine ≥ 10 μmol/L	
	β (95% CI)^a^	p	β (95% CI)^a^	p	β (95% CI)^a^	p
Univariate	-0.18 (-0.23; -0.13)	<0.01	-0.09 (-0.14; -0.03)	<0.01	-0.28 (-0.36; -0.20)	<0.01
Model 1	-0.16 (-0.21; -0.12)	<0.01	-0.9 (-0.14; -0.04)	<0.01	-0.27 (-0.35; -0.19)	<0.01
Model 2	-0.15 (-0.20; -0.11)	<0.01	-0.09 (-0.13; -0.04)	<0.01	-0.25 (-0.33; -0.18)	<0.01

25(OH)D, 25-hydroxyvitamin D; μmol/L, micromoles per liter; nmol/L, nanomoles per liter. β (95% CI), Beta coefficient with 95% confidence interval.

^a^ The β coefficients represent the change in homocysteine concentration in μmol/L between baseline and follow up per 25 nmol/L change in 25(OHD) concentration between baseline and follow up.

The β coefficients are calculated for those observations where the change in 25(OH)D concentrations was in the range of 0 to 75 nmol/L as the association of change in homocysteine and change in serum 25(OH)D concentrations is linear for this range (see Fig 1). As such, a total of 5,029 pairs of baseline and follow up visit were considered, 3,030 had baseline homocysteine concentrations <10 μmol/L, and 1,999 had baseline homocysteine concentrations ≥10 μmol/L. Model 1: adjusted for sex, baseline age (per 10 years), elevated homocysteine, body mass index, hypertension, serum LDL-cholesterol, smoking status, alcohol status, physical activity, and physical activity change during follow up. Model 2: adjusted for the same covariates as model 1, and additionally adjusted for serum vitamin B_12_ status at baseline and at follow up.

Table 4 presents the results from the longitudinal analyses of the risk of elevated homocysteine concentrations at follow up. The multivariable analysis revealed that relative to participants with baseline serum 25(OH)D concentrations less than 50 nmol/L, those with concentrations of “50–75”, “75–100”, “100–125”, and ≥125 nmol/L at baseline were 0.67 (95% confidence intervals: 0.45–0.98), 0.70 (0.44–1.09), 0.44 (0.23–0.84), and 0.39 (0.21–0.72) times as likely to have elevated homocysteine at follow up, respectively. With respect to the effect of temporal changes in serum 25(OH)D concentrations, relative to visits without temporal increases in 25(OH)D concentrations, follow up visits with increases of “<25”, “25–50”, “50–75”, “≥75” nmol/L at follow up were 0.92 (0.62–1.37), 0.52 (0.33–0.80), 0.34 (0.20–0.58), and 0.32 (0.19–0.54) times as likely to have elevated homocysteine, respectively. This effect of temporal changes in serum 25(OH)D concentrations on risk to for elevated homocysteine was similar in analyses restricted to participants with complete information on Vitamin B_12_. Vitamin B_12_ status was found to have an immediate impact on homocysteine concentrations, as adequate vitamin B_12_ measured at baseline did not influence the risk of elevated homocysteine concentrations at follow up, whereas adequate vitamin B_12_ at follow up significantly reduced the risk of elevated homocysteine concentrations at follow up [OR = 0.19 (0.11–0.32)]. Male gender, age, and smoking status were also significantly associated with serum homocysteine concentrations, while no significant associations were observed for hypertension and LDL cholesterol.

**Table 4:** Risk for elevated homocysteine^a^ at follow up among 4475 study participants with a total of 6605 follow up visits.

		Univariate		Multivariable^b^ not adjusted for vitamin B_12_		Multivariable^c^ adjusted for vitamin B_12_	
	#visits	OR (95% CI)	p	OR (95% CI)	p	OR (95% CI)	p
**Baseline 25(OHD), nmol/L**							
<50	1350	Reference		Reference		Reference	
50–<75	1982	0.55 (0.39–0.79)	<0.01	0.61 (0.41–0.89)	0.01	0.67 (0.45–0.98)	0.04
75–<100	1508	0.54 (0.37–0.78)	<0.01	0.61 (0.39–0.95)	0.03	0.70 (0.44–1.09)	0.11
100–<125	944	0.37 (0.23–0.59)	<0.01	0.39 (0.21–0.73)	<0.01	0.44 (0.23–0.84)	0.01
> = 125	821	0.35 (0.20–0.62)	<0.01	0.34 (0.18–0.64)	<0.01	0.39 (0.21–0.72)	<0.01
**Changes in 25(OH)D during follow up compared with baseline, nmol/L**							
No improvement	1277	Reference		Reference		Reference	
Increase of < 25	1530	1.26 (0.90–1.76)	0.19	0.82 (0.56–1.21)	0.31	0.92 (0.62–1.37)	0.68
Increase of 25–< 50	1480	0.86 (0.60–1.23)	0.40	0.48 (0.32–0.74)	<0.01	0.52 (0.33–0.80)	<0.01
Increase of 50–< 75	1017	0.60 (0.39–0.92)	0.02	0.29 (0.18–0.49)	<0.01	0.34 (0.20–0.58)	<0.01
Increase of > = 75	1301	0.67 (0.44–1.01)	0.06	0.28 (0.17–0.45)	<0.01	0.32 (0.19–0.54)	<0.01
**Baseline elevated homocysteine**	6605	16.6 (12.5–22.0)	<0.01	16.7 (12.1–23.0)	<0.01	15.7 (11.3–21.6)	<0.01
**Men vs. women**	6605	2.41 (1.77–3.27)	<0.01	1.65 (1.17–2.32)		1.82 (1.29–2.56)	<0.01
**Baseline age (per 10 years)**	6605	1.22 (1.11–1.34)	<0.01	1.17 (1.02–1.33)	0.02	1.26 (1.10–1.45)	<0.01
**BMI at baseline**^d^							
<18.5	60	1.51 (0.20–11.49)	0.69	1.44 (0.25–8.27)	0.68	1.53 (0.28–8.33)	0.62
18.5–<25.0	2014	Reference		Reference		Reference	
25.0–<30.0	2532	1.56 (1.11–2.20)	0.01	1.19 (0.81–1.74)	0.38	1.08 (0.74–1.58)	0.71
> = 30.0	1903	1.64 (1.16–2.31)	0.01	1.06 (0.72–1.57)	0.76	0.99 (0.67–1.46)	0.95
**Baseline hypertension**^e^							
Normal	4778	Reference		Reference		Reference	
Elevated	1234	1.55 (1.13–2.13)	0.01	1.08 (0.77–1.54)	0.65	1.10 (0.77–1.57)	0.61
**Baseline LDL-cholesterol**^f^							
Normal	2170	Reference		Reference		Reference	
Elevated	4144	1.33 (1.00–1.78)	0.05	1.02 (0.74–1.40)	0.90	0.98 (0.71–1.34)	0.88
**Baseline tobacco smoking**^d^							
Never smoker	1904	Reference		Reference		Reference	
Past smoker	880	1.62 (1.05–2.51)	0.03	1.31 (0.83–2.08)	0.25	1.26 (0.79–2.00)	0.33
Current smoker	526	3.03 (1.92–4.79)	0.00	2.63 (1.50–4.60)	<0.01	2.89 (1.62–5.15)	<0.01
**Baseline alcohol drinking**^d^							
Non-drinker	1392	Reference		Reference		Reference	
Drinker	1872	1.11 (0.75–1.65)	0.59	1.45 (0.96–2.21)	0.08	1.46 (0.95–2.23)	0.08
**Physical activity at baseline**^d^							
Low	1316	Reference		Reference		Reference	
Moderate	961	0.69 (0.45–1.08)	0.10	0.78 (0.49–1.25)	0.31	0.84 (0.52–1.35)	0.47
High	997	0.57 (0.36–0.90)	0.02	0.69 (0.41–1.19)	0.18	0.72 (0.41–1.24)	0.24
**Physical activity change**^d^							
No improvement	970	Reference		Reference		Reference	
Moderate improvement	641	1.34 (0.84–2.12)	0.22	1.36 (0.81–2.26)	0.24	1.32 (0.78–2.23)	0.30
High improvement	394	0.88 (0.46–1.69)	0.70	0.89 (0.40–1.95)	0.76	0.90 (0.40–2.00)	0.80
**Vitamin B12 status at baseline**^d^^,^ ^e^							
Deficiency/Insufficiency	328	Reference		-	-	Reference	
Adequate	3620	0.25 (0.16–0.38)	<0.01	-	-	0.89 (0.53–1.50)	0.66
**Vitamin B12 status at follow up**^d^^,^^g^							
Deficiency/Insufficiency	153	Reference		-	-	Reference	
Adequate	5765	0.11 (0.07–0.17)	<0.01	-	-	0.19 (0.11–0.32)	<0.01

25(OH)D, 25-hydroxyvitamin D; LDL-cholesterol, low-density lipoprotein cholesterol; OR (95% CI), odds ratio with 95% confidence interval; #visits, numbers of follow up visits; nmol/L, nanomoles per liter.

^a^ Elevated homocysteine was defined as serum concentrations >13 μmol/L.

^b^ The multivariable analysis adjusted for sex, baseline age (per 10 years), baseline elevated homocysteine, body mass index, hypertension, serum LDL-cholesterol, smoking status, alcohol status, physical activity, and physical activity change during follow up.

^c^ The multivariable analysis adjusted for the same confounders as above and additionally adjusted for vitamin B_12_ status.

^d^ Missing values were considered as ‘a missing category’ in the regression analyses.

^e^ Hypertension was defined as blood pressure ≥140/90 mm Hg, or taking antihypertensive medications.

^f^ Elevated LDL-cholesterol was defined as LDL-cholesterol concentration ≥2.6 mmol/L.

^g^ Vitamin B_12_ adequacy was defined as serum concentrations >220 picomoles per liter.

Of the 4276 participants with information on renal function, 108 (3%) had impaired renal function defined as Glomerular Filtration Rate (GFR) below 60 mL/min/1.73 m^2^ on consecutive visits. An analysis restricted to the 4168 participants with normal renal function (see S1 Table) revealed very similar associations as the analysis for the groups as whole (presented in Table 4). For participants with normal renal function, relative to follow up visits without temporal increases in 25(OH)D concentrations, follow up visits with increases of “<25”, “25–50”, “50–75”, “≥75” nmol/L were 0.97 (0.64–1.48), 0.57 (0.36–0.91), 0.35 (0.20–0.61), and 0.34 (0.20–0.59) times as likely to have elevated homocysteine, respectively (S1 Table).

Since homocysteine concentrations, on average, are higher in men than in women, we further considered sex specific thresholds for elevated homocysteine (14.2 for women and 15.2 for men). This analysis is included in S1 Table and revealed that relative to follow up visits without temporal increases in 25(OH)D concentrations, follow up visits with increases of “<25”, “25–50”, “50–75”, “≥75” nmol/L were 1.12 (0.61–2.04), 0.51 (0.26–0.98), 0.54 (0.25–1.18), and 0.32 (0.14–0.72) times as likely to have elevated homocysteine, respectively.

## Discussion

In the current longitudinal study of 4475 Canadians, we found that both higher serum 25(OH)D concentrations at baseline, as well as greater temporal improvements of 25(OH)D were associated with a reduction in the risk for elevated homocysteine.

Amer and Qayyum published the first epidemiological study that reported an inverse association between serum 25(OH)D and homocysteine concentrations using cross-sectional data from the NHANES [10]. They described this relationship as nonlinear, as it was only observed among those with serum 25(OH)D concentrations equal to or below 52.5 nmol/L. For every 25 nmol/L difference in 25(OH)D among those with 25(OH)D concentrations below 52.5 nmol/L and those with 25(OH)D above 52.5 nmol/L, homocysteine concentrations were 0.49 μmol/L lower (95% confidence intervals: -0.67; -0.31) and 0.02 μmol/L lower (-0.08; 0.11), respectively. We observed a similar strong association between 25(OH)D and homocysteine for 25(OH)D concentrations below 50 nmol/L. However, unlike the observation by Amer and Quyyan, we found this association also to be significant among those with 25(OH)D above 50 nmol/L. Differences between the two studies may be due to differences in study populations and lifestyles of the participants. Data used in the current study were from volunteers enrolled in a preventative health program. This sample is therefore likely more heterogeneous than that of the NHANES. It is also important to note that different thresholds for 25(OH)D were used. We had chosen 50 nmol/L or less as the threshold because this is often used in definitions of vitamin D deficiency and insufficiency [7, 8]. Nevertheless, both the study by Amer and Qayyum and ours revealed inverse associations between 25(OH)D and homocysteine concentrations in individuals of low vitamin D status. The present study extended the existing work on cross sectional associations with revealing associations between temporal changes in 25(OH)D and coinciding changes in homocysteine. These associations were stronger for individuals with above average homocysteine concentrations at baseline relative to those with below average concentrations at baseline.

We observed that the prevalence of elevated homocysteine decreased from 11.0% at baseline to 4.5% at follow up parallel with temporal increases in serum 25(OH)D. Our multivariable analysis confirmed that an temporal increase in 25(OH)D concentration independently predict a lower risk for elevated homocysteine at follow up. Since evidence has emerged that links elevated serum homocysteine with an increased risk of CVD, the present study may be of great importance from the point-of-view of public health and disease prevention. A meta-analysis examining the relationship between homocysteine and CVD estimated that the risk of ischemic heart disease was reduced by 16%, and that of stroke by 24% for every 3 μmol/L decrease of serum homocysteine [16]. Therefore, sun exposure and vitamin D supplementation effective in lowering homocysteine along with reductions in the consumption of alcohol and tobacco may decrease the risk for CVD. However, it should be noted that in a recent systematic review of randomized controlled trails of individuals at risk for or with established CVD, homocysteine-lowering interventions of treatment with B vitamins was not found to significantly reduce the risk of cardiovascular outcomes [17]. This review therefore suggests that homocysteine-lowering interventions are not effective in the prevention of CVD in at risk populations. However, the role of homocysteine-lowering interventions in the primary prevention of CVD (that is the prevention of healthy individuals to become at risk for CVD) is less established [10]. While the present study seems to show evidence for a homocysteine-lower effect with improvements in vitamin D status, the observed associations may also result from a mechanism whereby vitamin D changes are altering another factor that homocysteine is a marker for. This factor may or may not be a risk factor for cardiovascular disease. Several well-powered, long-term randomized controlled trials are currently underway that may establish the efficacy of vitamin D in the prevention of CVD and reveal the role of homocysteine [18].

Homocysteine is a sulfur amino acid that is metabolized in two ways: through the remethylation to methionine, and through the transsulfuration to cystathionine. The former pathway requires folate and vitamin B_12_, and the latter pathway requires vitamin B_6_ and cystathionine β-synthase (CβS) to catalyze serine. The underlying mechanism by which vitamin D acts on reducing serum homocysteine has not yet been elucidated. It is unknown whether vitamin D acts directly to lower homocysteine concentrations, or if vitamin D is a catalyst for facilitating other cofactors, such as vitamin B_12_, vitamin B_6_ and other enzymes involving in homocysteine metabolism. While the result of *in vitro* investigation of the effects of active vitamin D (1,25-dihydroxyvitamin D_3_) on CβS, an enzyme involved in the transsulfuration pathway for homocysteine disposal, suggest that increases in CβS may potentially explain the mechanism by which vitamin D contributes to the breakdown of homocysteine [19]. In the present study, we observed that vitamin D and vitamin B_12_ are independently associated with homocysteine concentrations, which suggests independent pathways. It has also been suggested that vitamin D supports folate and other cofactors in homocysteine disposal. We were not able to investigate the relationship between folate and vitamin D since the prevalence of folate deficiency in the Canadian population is less than 1% [20] due to the national acid folic fortification program of Canada [21, 22].

Currently, the definition for the normal range of homocysteine is not standardized, and is instead usually defined by an arbitrary cut-off above the 95^th^ percentile of the study population [23]. In the present study, we considered homocysteine concentrations >13 μmol/L as elevated in accordance with reports from CHMS of the general Canadian population [14]. At baseline, 11% of our participants had homocysteine concentrations above 13 μmol/L, which is higher than that of the CHMS which reported that 5% of Canadians between 6 to 79 years of age had serum homocysteine concentrations above 13 μmol/L [14]. Although differences in homocysteine metabolism between women and men have been reported [24], our sensitivity analysis that considered sex specific thresholds revealed very similar findings with respect to the effect of an improvement of vitamin D status on changes in the risk for elevated homocysteine concentrations.

The longitudinal design, large sample size and the wide range of serum 25(OH)D concentrations are strengths of the present study. A further strength of the study was our ability to adjust for serum vitamin B_12_, as this is an important factor in the homocysteine metabolism. As limitations we acknowledge that this study was conducted among volunteer participants of a preventive health program. These participants are not representative of the general population. The preventive health program not only encourages supplementation with vitamin D but rather healthy lifestyles in general. In this regard, we did report that physical active levels improved during follow up. We also observed a reduction of subjects with low levels of vitamin B_12_ which may results from better diets and multivitamin supplements. However, other lifestyle changes may also have occurred, though not recorded, such as for example a reduction in the consumption of red meat. These lifestyle changes may also have affected homocysteine concentrations. Other limitations relate to inaccuracies of the vitamin D assay and the fact that vitamin D was routinely collected and subjected to the assay rather than batch-wise. Missing values for the various confounding variables may have contributed to the inadequate accounting of their confounding potential. Particularly the number of missing values for vitamin B_12_ was high. However, our sub-analyses restricted to participants with complete information on vitamin B_12_ revealed very similar results. Lastly, participants differ in how they use the services of the preventive health program. Some would seek advise without biomarker assessment, some would only have these assessed at baseline whereas others would have these assessed annually. Of the 7579 participants with one or more follow up visits, 7533 had homocysteine and 25(OH)D assessments at baseline, and 4475 (59.4%) at baseline and at follow up. The latter group was included in the present analyses but appeared to have on average lower 25(OH)D and higher homocysteine concentrations as compared to those who had only baseline assessments. This further underlines that caution is warranted in the generalization of the present findings.

In summary, the present study is the first to reveal that prospective improvements in vitamin D status decreased the risk of elevated homocysteine concentrations. In Canada, 35% of adults are vitamin D deficient [25], in large part due to inadequate cutaneous synthesis of vitamin D. Therefore, population-based strategies aimed at improving vitamin D status and bone health may also contribute to lowering homocysteine concentrations, and potentially to the primary prevention of CVD.

## Supporting Information

S1 Table Risk for elevated homocysteine at follow up estimated through a sensitivity analysis that used sex specific threshold of homocysteine and through a sensitivity analysis restricted to participants with normal kidney function. (DOCX)
