# Supplementary material for: The Effect of Serum 25-Hydroxyvitamin D on Elevated Homocysteine Concentrations in Participants of a Preventive Health Program
Source: PLoS One. 2016 Aug 22;11(8):e0161368. doi: 10.1371/journal.pone.0161368 (PMC4993504; doi:10.1371/journal.pone.0161368)
Supplement: S1 Table — (DOCX) [file pone.0161368.s001.docx]

**S1 Table. Risk for elevated homocysteine at follow up estimated through a sensitivity analysis that used sex specific threshold of homocysteine and through a sensitivity analysis restricted to participants with normal kidney function**

|  |  | Multivariable model^a,c^ of a sensitivity analysis using sex specific threshold of homocysteine (n=4475) | |  | Multivariable model^b,c^ of a sensitivity analysis among participants with normal kidney function (n=4168) | |
| --- | --- | --- | --- | --- | --- | --- |
|  |  | OR (95% CI) | p |  | OR (95% CI) | p |
| **Baseline 25(OHD), nmol/L** |  |  |  |  |  |  |
| <50 |  | Reference |  |  | Reference |  |
| 50 - <75 |  | 0.46 (0.26-0.82) | 0.01 |  | 0.68 (0.45-1.01) | 0.06 |
| 75 - <100 |  | 0.67 (0.35-1.29) | 0.23 |  | 0.75 (0.46-1.21) | 0.24 |
| 100 - <125 |  | 0.46 (0.20-1.06) | 0.07 |  | 0.46 (0.23-0.91) | 0.02 |
| >=125 |  | 0.31 (0.13-0.78) | <0.01 |  | 0.36 (0.18-0.71) | <0.01 |
| **Changes in 25(OH)D during follow up compared with baseline, nmol/L** |  |  |  |  |  |  |
| No improvement |  | Reference |  |  | Reference |  |
| Increase of < 25 |  | 1.12 (0.61-2.04) | 0.71 |  | 0.97 (0.64-1.48) | 0.88 |
| Increase of 25 - < 50 |  | 0.51 (0.26-0.98) | 0.04 |  | 0.57 (0.36-0.91) | 0.02 |
| Increase of 50 - < 75 |  | 0.54 (0.25-1.18) | 0.12 |  | 0.35 (0.20-0.61) | <0.01 |
| Increase of >= 75 |  | 0.32 (0.14-0.72) | <0.01 |  | 0.34 (0.20-0.59) | <0.01 |
| **Baseline elevated homocysteine** |  | 29.5 (18.0-48.2) | <0.01 |  | 15.3 (10.9-21.6) | <0.01 |
| **Men vs. women** |  | 0.87 (0.55-1.39) | 0.57 |  | 2.02 (1.39-2.92) | <0.01 |
| **Baseline age (per 10 years)** |  | 1.08 (0.88-1.31) | 0.47 |  | 1.16 (1.01-1.33) | 0.04 |
| **BMI at baseline^d^** |  |  |  |  |  |  |
| <18.5 |  | 2.43 (0.48-12.27) | 0.28 |  | 1.89 (0.34-10.31) | 0.46 |
| 18.5 - <25.0 |  | Reference |  |  | Reference |  |
| 25.0 - <30.0 |  | 0.75 (0.43-1.31) | 0.31 |  | 1.13 (0.75-1.71) | 0.55 |
| >=30.0 |  | 0.67 (0.37-1.21) | 0.18 |  | 0.96 (0.63-1.48) | 0.86 |
| **Baseline hypertension^e^** |  |  |  |  |  |  |
| Normal |  | Reference |  |  | Reference |  |
| Elevated |  | 1.32 (0.76-2.30) | 0.32 |  | 1.29 (0.89-1.88) | 0.18 |
| **Baseline LDL-cholesterol^f^** |  |  |  |  |  |  |
| Normal |  | Reference |  |  | Reference |  |
| Elevated |  | 0.78 (0.50-1.21) | 0.26 |  | 0.98 (0.70-1.39) | 0.93 |
| **Baseline tobacco smoking^d^** |  |  |  |  |  |  |
| Never smoker |  | Reference |  |  | Reference |  |
| Past smoker |  | 2.04 (0.95-4.37) | 0.07 |  | 1.29 (0.78-2.12) | 0.32 |
| Current smoker |  | 3.71 (1.46-9.38) | <0.01 |  | 2.70 (1.50-4.87) | <0.01 |
| **Baseline alcohol drinking^d^** |  |  |  |  |  |  |
| Non-drinker |  | Reference |  |  | Reference |  |
| Drinker |  | 0.93 (0.48-1.82) | 0.83 |  | 1.40 (0.89-2.19) | 0.14 |
| **Physical activity at baseline^d^** |  |  |  |  |  |  |
| Low |  | Reference |  |  | Reference |  |
| Moderate |  | 0.55 (0.24-1.23) | 0.14 |  | 0.96 (0.58-1.60) | 0.89 |
| High |  | 0.73 (0.31-1.70) | 0.46 |  | 0.77 (0.43-1.38) | 0.38 |
| **Physical activity change^d^** |  |  |  |  |  |  |
| No improvement |  | Reference |  |  | Reference |  |
| Moderate improvement |  | 1.50 (0.72-3.12) | 0.28 |  | 1.41 (0.81-2.46) | 0.23 |
| High improvement |  | 0.93 (0.33-2.60) | 0.89 |  | 0.98 (0.42-2.26) | 0.96 |
| **Vitamin B12 status at baseline^d, e^** |  |  |  |  |  |  |
| Deficiency/Insufficiency |  | Reference |  |  | Reference |  |
| Adequate |  | 1.59 (0.76-3.36) | 0.22 |  | 0.88 (0.51-1.52) | 0.66 |
| **Vitamin B12 status at follow up^d,g^** |  |  |  |  |  |  |
| Deficiency/Insufficiency |  | Reference |  |  | Reference |  |
| Adequate |  | 0.13 (0.07-0.27) | <0.01 |  | 0.17 (0.10-0.30) | <0.01 |

25(OH)D, 25-hydroxyvitamin D; LDL-cholesterol, low-density lipoprotein cholesterol; OR (95% CI), odds ratio with 95% confidence interval; #visits, numbers of follow up visits; nmol/L, nanomoles per liter.

^a^ In this model, elevated homocysteine was defined as serum concentrations >14.2 µmol/L in women, and >15.2 in men corresponding to 95 percentile of sex specific baseline homocysteine distribution.

^b^ In this model, elevated homocysteine was defined as serum concentrations >13 µmol/L, applied to both sexes, and includes analysis restricted to 4168 paticipants who had normal renal functions defined as glomerular filtration rate at or greater than 60 mL/min/1.73 m^2^.

^c^ The multivariable analysis adjusted for sex, baseline age (per 10 years), baseline elevated homocysteine, body mass index, hypertension, serum LDL-cholesterol, smoking status, alcohol status, physical activity, and physical activity change during follow up, and vitamin B_12_ status.

^d^ Missing values were considered as ‘a missing category’ in the regression analyses.

^e^ Hypertension was defined as blood pressure ≥140/90 mm Hg, or taking antihypertensive medications.

^f^ Elevated LDL-cholesterol was defined as LDL-cholesterol concentration ≥2.6 mmol/L.

^g^ Vitamin B_12_ adequacy was defined as serum concentrations >220 picomoles per liter.
